# A preliminary study of suppression of candida infection by miconazole mucoadhesive tablets in oral or oropharyngeal cancer patients undergoing radiotherapy

**DOI:** 10.1038/s41598-022-14269-9

**Published:** 2022-06-17

**Authors:** Sakiko Soutome, Mitsunobu Otsuru, Yumiko Kawashita, Masako Yoshimatsu, Madoka Funahara, Maho Murata, Takashi Ukai, Masahiro Umeda, Toshiyuki Saito

**Affiliations:** 1 Department of Oral Health, Nagasaki University Graduate School of Biomedical Sciences, 1-7-1 Sakamoto, Nagasaki, 852-8588 Japan; 2 Department of Clinical Oral Oncology, Nagasaki University Graduate School of Biomedical Sciences, 1-7-1 Sakamoto, Nagasaki, 852-8588 Japan; 3 Oral Management Center, Nagasaki University Hospital, 1-7-1 Sakamoto, Nagasaki, 852-8501 Japan; 4 School of Oral Health Sciences, Faculty of Dentistry, Kyushu Dental University, 2-6-1 Manazuru, Kokurakitaku, Kitakyushu, Fukuoka 803-8580 Japan

**Keywords:** Cancer, Oncology

## Abstract

Elevated numbers of candida in the oral cavity often lead to oral candidiasis development in patients undergoing radiotherapy for oral or oropharyngeal cancer. This study aimed to verify the effect of miconazole mucoadhesive tablets on suppression of oral candida infection during radiotherapy. For this preliminary interventional study, miconazole mucoadhesive tablets were attached to the oral mucosa for 14 days from when grade 2 oral mucositis appeared in patients with oral or oropharyngeal cancer receiving radiotherapy, and the incidence of oral candidiasis was investigated. Various clinical factors were examined; univariate and multivariate Cox regression analyses were performed to investigate and compare the efficacy of this drug in preventing oral candidiasis with results of our previous study as historical control. Miconazole mucoadhesive tablets were administered to 18 patients, and oral candidiasis was observed in one patient (5.6%) after treatment completion. Among 144 historical control patients, 43 (29.9%) developed oral candidiasis. Multivariate Cox regression showed that miconazole mucoadhesive tablets significantly reduced oral candidiasis development during radiotherapy (*p* = 0.049, Hazard ratio 0.136, 95% confidence interval 0.019–0.994). This preliminary study suggests the efficacy of miconazole mucoadhesive tablets in preventing oral candidiasis in oral or oropharyngeal cancer patients treated with radiotherapy.

**Trial registration**: Japan Registry of Clinical Trials (jRCT), jRCTs071190023. Registered 3 September, 2019.

## Introduction

Oral mucositis is inevitable when radiotherapy (RT) is performed for treatment of head and neck cancers, whereby the oral cavity is exposed to radiation. Kawashita et al. demonstrated that grade 2 oral mucositis occured in more than 90% of oral cancer patients who required dietary changes due to moderate pain. In addition, grade 3 oral mucositis occured in 14% of those receiving RT alone, and in 34% of those receiving chemo-radiotherapy (CRT) that made oral feeding difficult due to severe oral mucositis^1^. Oral mucositis not only decreases patients’ quality of life (QOL), but can also lead to RT interruption and may even affect prognosis.

At present, there are few effective methods for the prevention and treatment of RT-induced oral mucositis^2,3^. In Japan, the efficacy and safety of steroid ointments (dexamethasone ointment and triamcinolone ointment) were initially reported in the 1980s; however, the use of steroid ointment during RT has remained controversial in the intervening years due to concerns that the use of steroid ointments can make oral candidiasis more likely to occur. We examined oral candidiasis during RT and reported that candidiasis occurs in about 30% of patients undergoing RT, and that the use of steroid ointment does not promote the development of oral candidiasis^4,5^. Upon development of oral candidiasis, the use of an antifungal agent is recommended and steroid ointments are best avoided. Further discontinuation of steroid ointment application due to the development of oral candidiasis leads to a decrease in the QOL of the patient. Hence, it is important to prevent oral candidiasis during RT.

Oravi^®^ is a miconazole mucoadhesive tablet that has been recently developed for the treatment of oral candidiasis. The purpose of this study was to examine whether the development of oral candidiasis during RT can be suppressed by prophylactic administration of this drug from the time when the symptoms associated with grade 2 oral mucositis first appeared.

## Results

### Patient characteristics

Eighteen patients were enrolled in the intervention group. All patients were administered miconazole mucoadhesive tablets without any reported side effects. The demographic and treatment-related factors of the patients are summarized in Table 1. The enrolled patients comprised 15 male and three female patients, with an average age of 60.7 years (range 40–82 years). The primary site of involvement was the oral cavity in 11 patients and the oropharynx in seven. All 18 patients received IMRT with an average total dose of 65.0 Gy. Thirteen patients were administered concomitant cisplatin and one was administered concomitant cetuximab. All 18 patients developed oral mucositis of grade 2–3 during RT.

**Table 1:** Patient characteristics of the intervention and control groups.

Variable	Intervention group (n = 18)	Control group (n = 144)	*p* value
**Age (year)**			
Mean ± SD	60.7 ± 12.4	66.9 ± 10.7	0.023
**Sex**			
Male	15	103	0.403
Female	3	41	
**Primary site**			
Oral cavity	11	95	0.794
Oropharynx	7	49	
**RT method**			
3D-CRT	0	109	< 0.001
IMRT	18	35	
**Concomitant therapy**			
RT alone	4	37	0.211
CRT	13	76	
BRT	1	30	
Total RT dose (Gy)	65.0 ± 5.15	62.9 ± 5.20	0.112
Minimum leukocyte count during RT (10^3^/µL)	2.69 ± 1.55	3.55 ± 2.15	0.100
**Oral mucositis**			
Grade 0–1	0	29	0.045
Grade 2–3	18	115	
**Steroid ointment**			
(−)	4	54	0.297
(+)	14	90	

A total of 144 patients were enrolled in the historical control group. There were some differences between the intervention and historical control groups. Patients in the intervention group were significantly younger, had more IMRT, and had a higher incidence of severe oral mucositis than those in the control group.

### Oral candidiasis development and related factors

**Figure 1:**
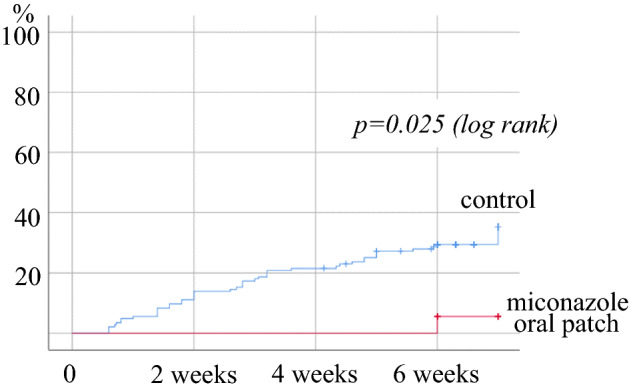
Relationship between the administration of miconazole mucoadhesive tablets and the development of oral candidiasis.

Oral candidiasis developed in one of the 18 patients (5.6%) in the intervention group and in 43 of the 144 patients (29.9%) in the control group. The patient in the intervention group who developed candidiasis did so after the end of miconazole administration. The Kaplan–Meier curve showed significantly lower incidence rate in the intervention group than in the control group (log rank test: *p* = 0.025) (Fig. 1).

Univariate Cox regression analysis showed that prophylactic administration of miconazole mucoadhesive tablet tended to suppress the development of oral candidiasis; however, there was no significant difference between the two groups (*p* = 0.055) (Table 2). Multivariate analysis was performed by including the primary site of involvement, leukocyte count, and oral mucositis, which together were found to be associated with the onset of candidiasis in a previous study^3^; this analysis found that only prophylactic administration of miconazole mucoadhesive tablets significantly suppressed the development of oral candidiasis (*p* = 0.049, Hazard ratio 0.136, 95% confidence interval 0.019–0.994) (Table 3).

**Table 2:** Factors related to the development of oral candidiasis (univariate cox regression analysis).

Variable	*p* value	HR	95% CI
Age (year)	0.305	1.014	0.987–1.041
**Sex**			
Female/male	0.408	0.766	0.407–1.441
**Primary site**			
Oropharynx/oral cavity	0.376	0.752	0.396–1.428
**RT method**			
IMRT/3D-CRT	0.062	0.514	0.256–1.034
**Concomitant therapy**			
CRT or BRT/RT alone	0.357	0.815	0.526–1.260
Total RT dose (Gy)	0.436	0.977	0.922–1.036
Minimum leukocyte count during RT (10^3^/µL)	0.405	1.067	0.916–1.242
**Oral mucositis**			
Grade 2–3/0–1	0.369	1.483	0.628–3.505
**Steroid ointment**			
(+)/(−)	0.148	1.629	0.841–3.154
**Administratin of miconazole patch**			
(+)/(−)	0.055	0.144	0.020–1.044

**Table 3:** Factors related to the development of oral candidiasis (multivariate cox regression analysis).

Variable	*p* value	HR	95% CI
**Primary site**			
Oropharynx/oral cavity	0.350	0.735	0.386–1.401
Minimum leukocyte count during RT (10^3^/µL)	0.611	1.043	0.886–1.229
**Oral mucositis**			
Grade 2–3/0–1	0.193	1.774	0.749–4.205
**Administratin of miconazole patch**			
(+)/(−)	0.049	0.136	0.019–0.994

## Discussion

This preliminary study suggests that prophylactic administration of miconazole mucoadhesive tablets could potentially prevent the development of oral candidiasis during RT in oral or oropharyngeal cancer patients.

Head and neck cancer patients undergoing RT often develop oral candidiasis over the course of their treatment. Soysa et al. stated that RT and/or cytotoxic chemotherapy act on the oral mucosa and bone marrow and cause ulceration of the oral mucosa and leukocytopenia. Further, they found that patients receiving antibiotics show an increased susceptibility to candidiasis due to changes associated with *Candida albicans* flora that in turn may cause an increase in the number of *C. albicans* spores^6^. A retrospective analysis of 326 patients found that leukocytopenia and oral mucositis of grade 2 or more were the most common risk factors associated with oral candidiasis in RT patients^5^. These findings suggest that it is important to prevent leukopenia and oral mucositis in order to reduce the risk of developing oral candidiasis, but in many cases these are unavoidable adverse events associated with RT and chemotherapy. Therefore, it is necessary to consider preventing an increase in the number of candida spores arising in the oral mucosa of patients undergoing RT.

Several researchers have reported on changes in the number of candida spores present during and after RT. Arrifin et al. reported that *Candida albicans* counts significantly increased after 24 weeks of RT^7^. Suryawanshi reported that the number of *Candida albicans* spores increased during RT, and advocated that prophylactic antifungal treatment should be given to patients showing development of oral mucositis or dry mouth^8^. Of note, we also quantified total bacteria and *Candida albicans* in saliva by real-time polymerase chain reaction (PCR) during RT in oral or oropharyngeal cancer patients, and found that the number of total bacteria did not change but the number of *Candida albicans* increased significantly during RT^9^.

Zhang et al. reported the efficacy of miconazole for the treatment of oral candidiasis by a systematic review and meta-analysis, and stated that miconazole oral gel may be more effective than other formulations with regard to long-term results^10^. Oral candidiasis can be cured in a relatively short period of time by topical administration of antifungal agents such as miconazole oral gel and amphotericin B syrup. However, during the onset of symptoms, in addition to the pain and discomfort caused by candidiasis, an exacerbation of oral mucositis might be seen in patients who refrain from the use of steroid ointment; this is particularly problematic because steroid ointment is a therapeutic agent for radiation stomatitis. Therefore, prevention of oral candidiasis is important for maintaining patients’ QOL during RT.

Recently developed miconazole mucoadhesive tablets have shown a similar efficacy to miconazole oral gel on oral candidiasis and have a lower risk of side effects due to the tablets’ efficacy at lower doses compared to miconazole gel^11^. In addition, this regimen has excellent medication compliance and has been shown to have less discomfort during use than does the gel type. However, miconazole has many contraindicated drugs, thus potentially limiting its clinical use.

We have previously shown by real-time PCR analysis that candida growth in the saliva can be suppressed by administration of miconazole mucoadhesive tablets for 14 days in patients with Head and Neck cancer undergoing RT^9^. Therefore, in this study, we decided to preliminarily examine the safety and effect of miconazole mucoadhesive tablets on suppression of oral candida infection. This study found that only one of 18 cases developed oral candidiasis after the administration of miconazole mucoadhesive tablets was completed, and that no patients in the intervention group developed oral candidiasis during the course of miconazole administration; this suggests that miconazole mucoadhesive tablets had an effect on suppression of candida infection.

As this was a one-arm study, we compared its findings with the incidence of oral candidiasis in patients with oral or oropharyngeal cancer receiving RT, which was previously examined in our department. The historical control group includes cases from a previous period, and there were some differences in background factors from patients in this study, such as the irradiation method being used in the control group was primarily three-dimensional conformal radio-therapy. As a result, we showed that the development of oral candidiasis was significantly suppressed in this study compared to the historical control group. These findings seem to indicate the effect of miconazole mucoadhesive tablets on suppression of oral candida infection in patients with oral or oropharyngeal cancer undergoing RT.

This study has some limitations. First, this is a preliminary study with a small number of patients, so it is unclear whether the results obtained can be generalized. Second, there may be a bias between the intervention and control groups because the study used historical controls rather than a randomized controlled trial. Third, in this preliminary study, oral candidiasis was diagnosed only by clinical findings, although it is generally diagnosed by both clinical findings and microbiological examination. In future randomized controlled trials, we plan to evaluate not only clinical findings but also culture tests. However, this study is the first to suggest that miconazole mucoadhesive tablets can safely and effectively prevent oral candidiasis in patients with oral or oropharyngeal cancer undergoing RT. We plan to conduct a large-scale randomized controlled trial in the future.

In conclusion, Miconazole mucoadhesive tablets attached to the oral mucosa for 14 days from the time when symptoms associated with grade 2 oral mucositis first appeared were effective in preventing oral candidiasis in patients with oral or oropharyngeal cancer receiving RT.

## Methods

### Study design

This was a preliminary, one-armed, prospective, interventional study to verify the effect of miconazole mucoadhesive tablets on suppression of oral candida infection during RT.

### Patients

Oral or oropharyngeal cancer patients aged 20–90 years who received RT at Nagasaki University Hospital between April 2020 and September 2021 were included in this study. Patients who received RT of 40 Gy or less were excluded participating in the study, as were those patients who received the following drugs that are contraindicated in combination with miconazole: warfarin potassium, pimozide, quinidine, triazolam, simvastatin, azernidipine, nisoldipine, blonanserin, rivaroxaban, asunaprevir, lomitapidomesyl, sildenafil, alprazolam, mitazolam, brotyrolam, methylprednisolone, selegiline, ebastin, imatinib mesylate, disopyramide, silostazole, and HIV protease inhibitor.

### Ethics approval and consent to participate

Ethical approval from the Clinical Research Review Board in Nagasaki University was obtained, and the study conformed to the tenets of the Declaration of Helsinki. This study was approved by the Clinical Research Review Board of Nagasaki University (No. CRB19-004) and registered at the Japan Registry of Clinical Trials (jRCT) on 03/09/2019 (jRCTs071190023). Details are available at the following address: https://jrct.niph.go.jp/re/reports/detail/3338. All participants was required to provide written informed consent. A copy of the consent form is included in the supplementary material.

### Intervention

In addition to the usual oral care that included oral hygiene instructions, removal of dental plaque and calculus, and gargling instructions provided by dentists and dental hygienists, a miconazole mucoadhesive tablet was applied to the oral mucosa (equivalent to canine fossa) once daily from the day that grade 2 oral mucositis was diagnosed. The tablet was administered for 14 days or until the end of RT. If oral candidiasis developed, the observation was terminated; in such cases, appropriate oral candidiasis treatment was then given at the discretion of the treating dentist.

### Variables

The following factors were examined: sex; age; primary tumor site; minimum leukocyte count during RT; RT method (three-dimensional conformal radiotherapy: 3D-CRT/intensity-modulated radiation therapy: IMRT); concomitant therapy (RT alone, chemo-radiotherapy: CRT, bio-radiotherapy: BRT); total RT dose; use of steroid ointment; oral mucositis (grade 0–1/grade 2–3); and oral candidiasis.

Diagnosis of oral candidiasis was based on the presence of clinical signs as determined by the head and neck oncologist or dentists using clinical criteria and did not depend on culture test positivity or negativity.

### The desired sample size, reasons for the sample size selected, and the actual enrolled number

Initially, this study was planned as a multicenter study of three institutions. As a result of our previous study, in 144 patients with oral and oropharyngeal cancer undergoing radiotherapy, oral candidiasis occurred in 31% patients. The effectiveness of miconazole mucoadhesive tablets in patients with oral candidiasis is reported to 46.8%. Although the suppressive effect of miconazole mucoadhesive tablets on development of oral candida infection have not been reported, the incidence rate of oral candidiasis in the intervention group is assumed to be 16%, and if α = 0.20 and power = 0.80, the number of cases required is 57 patients. About 10% cases are assumed to be dropped out, and the target number of cases is 64 cases. However, due to COVID-19 infection, it became impossible to hold meetings between facilities (online meetings were not common in Japan at the beginning), so it was unavoidable to register cases at a single facility at Nagasaki University, and we decided to reduce the target number cases to about 1/3, and finally only 18 cases were registered during the scheduled period.

### Statistical analysis

As a preliminary trial, this study examined the suppressive effect and safety of miconazole mucoadhesive tablets on oral candidiasis during RT in oral or oropharyngeal cancer patients. The primary endpoint is presence/absence and timing of onset of oral candidiasis. Data pertaining to patients with oral or oropharyngeal cancer were extracted from our previous observational study^4^ and used as historical controls; we subsequently made comparisons between these control data and the results obtained in this study. The historical controls have the same inclusion criteria as in this study, except for the exclusion criteria for miconazole contraindicated drugs. Patients in the historical control group are also patients in the same hospital as in this study, and it is considered that there is no major difference in background factors. However, there are some differences in treatment policies, such as the fact that IMRT has recently become more frequent as an irradiation method and that the combination with cetuximab has decreased and the combination with high-dose cisplatin has increased. The incidence of oral candidiasis between in 18 patients of the intervention group and in 144 patients in the historical control groups was assessed by the Kaplan–Meier method, and the factors affecting the development of oral candidiasis were analyzed by univariate and multivariate Cox regression analyses.

All statistical analyses were performed using SPSS software (version 26.0; Japan IBM Co., Tokyo, Japan). In all analyses, two-tailed p values < 0.05 were considered statistically significant.

## Data Availability

The data that support the findings of this study are available from the corresponding author upon reasonable request.
